# Metoidioplasty: Surgical Options and Outcomes in 813 Cases

**DOI:** 10.3389/fendo.2021.760284

**Published:** 2021-10-13

**Authors:** Noemi Bordas, Borko Stojanovic, Marta Bizic, Arpad Szanto, Miroslav L. Djordjevic

**Affiliations:** ^1^ Department of Urology, Semmelweis Hospital, Kiskunhalas, Hungary; ^2^ Belgrade Centre for Urogenital Reconstructive Surgery, Belgrade, Serbia; ^3^ School of Medicine, University of Belgrade, Belgrade, Serbia; ^4^ Urology Clinic, University of Pecs, Pecs, Hungary; ^5^ Department of Urology, Icahn School of Medicine at Mount Sinai, New York, NY, United States

**Keywords:** gender affirmation surgery, metoidioplasty, phalloplasty, urethroplasty, phalloplasty complications

## Abstract

**Introduction:**

Metoidioplasty is a variant of phalloplasty for transmen that includes the creation of the neophallus from a hormonally enlarged clitoris, urethral lengthening and scrotoplasty. The procedure results in male appearance of genitalia, voiding in standing position and preserved sexual arousal, but without possibility for penetrative intercourse. We evaluated outcomes of metoidioplasty at our center, based on latest surgical refinements.

**Methods:**

During the period of 14 years (from February 2006 to April 2020), 813 transmen with mean age of 24.4 years and mean body mass index of 24.6, underwent one stage metoidioplasty. Hysterectomy was simultaneously performed in 156, and mastectomy in 58 cases. Hysterectomy, mastectomy and metoidioplasty were done as a one-stage procedure in 46 transmen. Patients are divided in 5 groups, depending on the type of urethroplasty. Postoperative questionnaires were used to evaluate cosmetic and functional outcomes, as well as patients’ satisfaction.

**Results:**

Follow-up ranged from 16 to 180 months (mean 94 months). Mean surgery time was 170 minutes and mean hospital stay was 3 days. Length of the neophallus ranged from 4.8 cm to 10.2 cm (mean 5.6 cm). Urethroplasty was complication-free in 89.5% of cases, and ranged between 81% to 90.3% in different groups. Urethral fistula and stricture occured in 8.85% and 1.70% of cases, respectively. Other complications included testicular implant rejection in 2%, testicular displacement in 3.20% and vaginal remnant in 9.60% of cases. From 655 patients who answered the questionnaire, 79% were totally satisfied and 20% mainly satisfied with the result of surgery. All patients reported voiding in standing position and good sexual arousal of the neophallus, without possibility for penetrative intercourse due to small size of the neophallus.

**Conclusion:**

Metoidioplasty has good cosmetic and functional outcomes, with low complication rate and high level of patients’ satisfaction. In transmen who request total phalloplasty after metoidioplasty, all available phalloplasty techniques are feasable.

## Introduction

The prevalence rate of gender dysphoria can not be determined precisely, but estimations range between 5-14 per 100.000 and 2-3 per 100.000 for persons assigned male at birth (AMAB) and assigned female at birth (AFAB), respectively. Recent studies suggest that the prevalence of a self-reported transgender identity in children, adolescents and adults ranges from 500 to 1300 per 100.000, markedly higher than prevalence rates based on clinic-referred samples of adults (1–3). The World Professional Association for Transgender Health (WPATH) has established the Standards of Care (SOC), the guidelines for the multidisciplinary treatment of such individuals (4). The treatment consists of diagnostic assessment, psychotherapy, hormonal therapy, and surgical therapy. Psychiatric assessment is the first step and is very complex because it is necessary to confirm gender dysphoria. The next step is hormonal therapy, which evidently improves emotional well-being and social functioning (5). Some individuals decide to continue to gender affirmation surgery (GAS), which presents the last and irreversible step in a transition process. According to SoC guideliness, individuals who wish to undergo GAS are required to provide two recommendation letters from certified psychiatrists and a gender specialist, as well as a confirmation of having been on hormonal therapy prescribed by an endocrinologist for a period of a minimum of one year.

With the increasing prevalence of transgender adults, including persons with binary and non-binary identities, the number of gender affirmation surgery (GAS) has also risen throughout the world. Heterogenity within the trans group and different requirements regarding the requests for GAS pose a question of additional analysis regarding the patient wishes, expectations and limitations of available surgical approaches. Non-binary transgenders may require only partial genital reconstruction that will satisfy their needs of standing micturition, sexual function and esthetic appearance (6, 7). After 50 years of surgical experience and numerous modifications of techniques, neophalloplasty still presents a greatest challenge in GAS (8). Metoidioplasty is a variant of phalloplasty that includes the creation of the small-sized neophallus from a hormonally hypetrophied clitoris, urethral lenthening, perineoplasty and scrotoplasty. The results are male-like genitalia, voiding in standing position and preserved sexual arousal, but without possibility for penetrative intercourse. Additional advantages are full erogenous sensation, the ability of erection and minimal scarring of the donor-site in a single stage procedure. Clear understanding of the anatomy of the clitoris and its surrounding tissues are essential for performing successful metoidioplasty in transmen (9). Several authors have attempted to use the hypertrophied clitoris for neophalloplasty, along with urethroplasty in the past decades (10–16). The technique was first mentioned in 1973 by Durfee and Rowland, while the term metoidioplasty was used first by Laub (10, 11). Laub performed a staged procedure, but voiding in a standing position was not achieved after the first stage. Bouman reconstructed the urethra until the tip of the neoglans without ventral chordee release (12). Hage combined the techniques of Bouman and Laub to obtain voiding while standing (13). Lebovic and Laub in 1999 reported good results in the appearance of external genitalia, but the neophallus was usually small and curved because of the intact urethral plate (14). Later in a long-term follow-up study Hage and van Turnhout stated that an average 2.6 procedures are needed to achieve satisfying outcomes after metoidioplasty (15). A new modification was published by Perovic and Djordjevic with higher success rates using tubularized flaps for urethral reconstruction in 2003 (16). Wide experience with surgical reconstruction of hypospadias improved the technique futher which resulted in the latest use of combined buccal mucosa or genital skin grafts with different local genital flaps to achieve a successful result. Latest advances in surgical techniques and perioperative care enabled safe one-stage gender affirmation surgery, metoidioplasty with hysterectomy and mastectomy (17, 18).

The aim of this study is to evaluate outcomes and complications in 813 cases of metoidioplasty in transmen, focusing on our modifications of urethroplasty techniques.

## Materials and Methods

**Figure 1:**
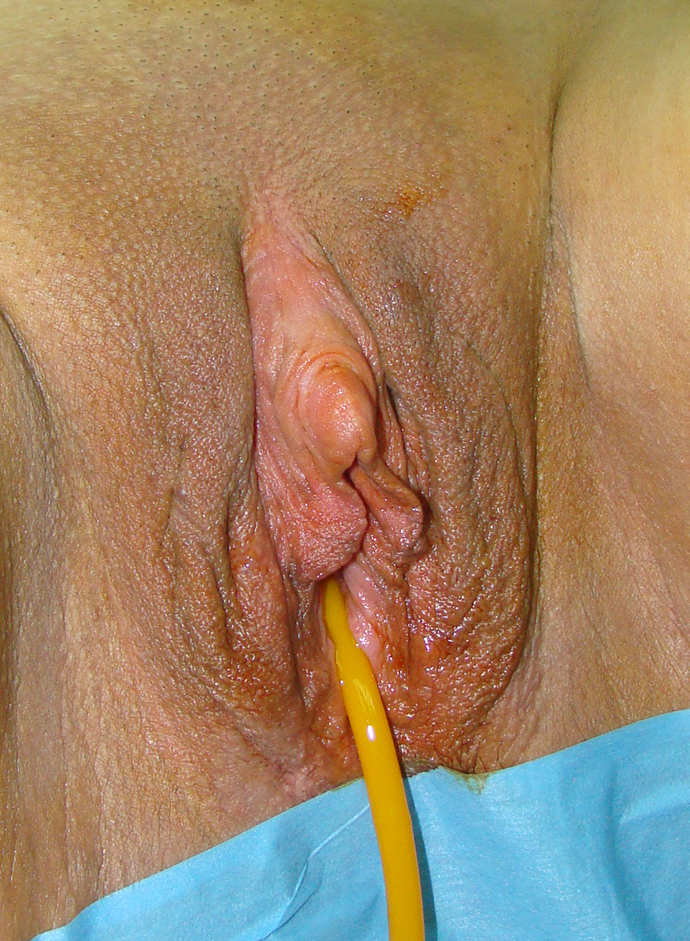
Preoperative appearance of female genitalia after topical testosterone treatment.

Between February 2006 and April 2020, a total of 813 transmen with a mean age of 24.4 years (range from 18 to 58 years) underwent single stage metoidioplasty with urethral lengthening. Mean body mass index in this group was 24.6 (ranged from 16.4 to 32.8). Inclusion criteria of latest version of WPATH Standards of Care were used in this study (4). The study protocol was approved by the Ethics Committee of Belgrade Center for Urogenital Reconstructive Surgery (approval number: 2020/11). Surgery was planned following cross-hormonal therapy, lasting a minimum of one year. Mean period of preoperative hormonal therapy was 32 months, and raged between 14 months and 24 years. Patients had undergone metoidioplasty simultaneously with hysterectomy and adnexectomy or mastectomy in 156 and 58 cases, respectively. Hysterectomy and mastectomy were performed simultaneously with metoidioplasty (one-stage GAS) in 46 out of 813 cases (5.6%) (**Table 1**). Complete metoidioplasty was performed in each case and included: vaginectomy, clitoral reconstruction, urethral lengthening, scrotoplasty and bilateral implantation of the testicular prostheses. Patients are divided in groups, depending on the type of urethroplasty: group A - tubularization urethroplasty (92 cases), group B - onlay flap urethroplasty either with dorsal clitoral or labia minora skin flaps (42 cases), group C - buccal mucosa graft (BMG) with clitoral skin flap urethroplasty (83 cases), group D - BMG with labia minora flap urethroplasty (537 cases), group E - labia minora skin graft with clitoral skin/labia minora flap urethroplasty (59 cases). An optimal technique of urethroplasty in each case depends on individual anatomical characteristics and patients’ preference for buccal mucosa grafting. Patients were treated preoperatively with topical dihydrotestosterone gel twice daily for three months prior to surgery, in order to obtain clitoral enlargement. The same regimen as for hypospadias is used, with well defined benefits (19). The clitoris had been additionally enlarged using vacuum device during the 6-month period leading up to surgery (**Figure 1**).

**Table 1:** Patients’ demographic characteristics and type of GAS.

Patients	Age (mean)	Follow-up (mean)	Preoperative hormonal th (mean)	Gender affirmation surgery (GAS)			
				Metoidioplasty	Metoidioplasty+ hysterectomy	Metoidioplasty+ mastectomy	One-stage GAS*
813	18-58y (24.4y)	16-180m (94m)	14m-24y (32m)	553	156	58	46

*One-stage GAS: hysterectomy + mastectomy + metoidioplasty.

### Operative Technique

**Figure 2:**
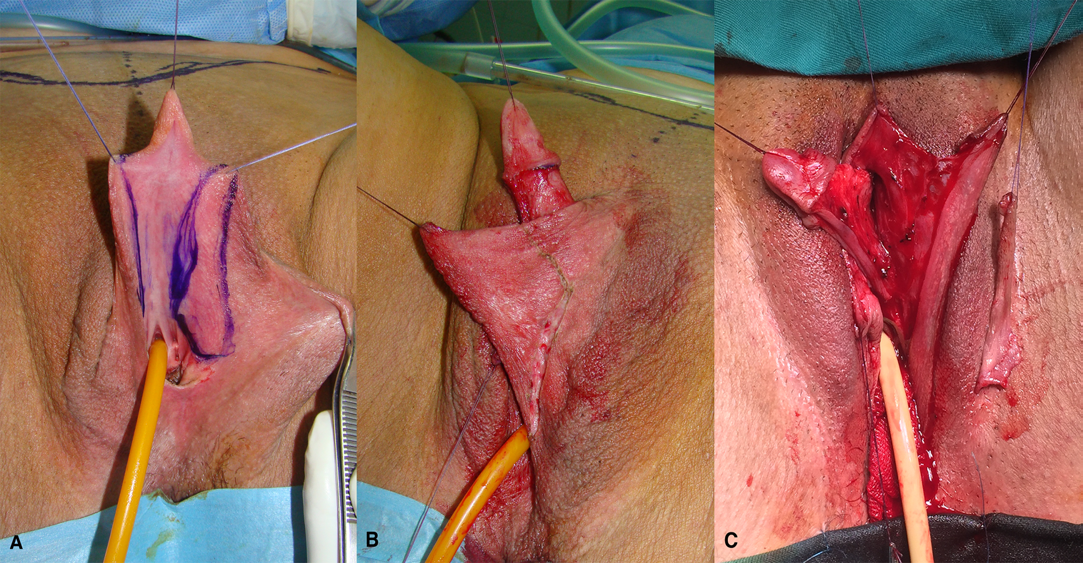
Drawings of the urethral plate and inner side of labia minora, which is planned for urethral reconstruction **(A)**; outer layer of the labia minora is planned for skin graft harvesting **(B)**; labia minora flap and skin graft are created for urethral lengthening **(C)**.

**Figure 3:**
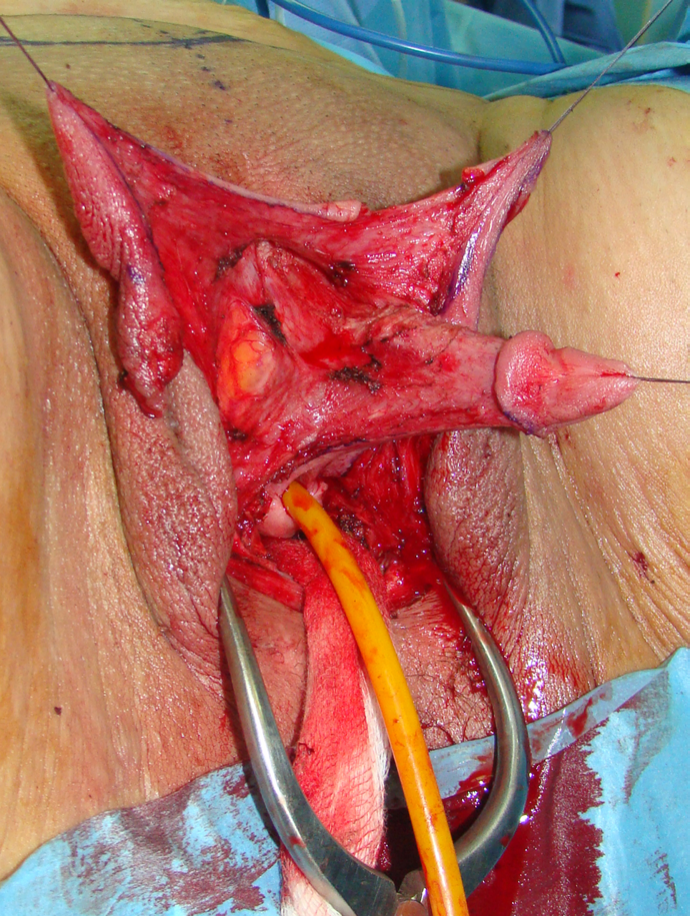
Clitoris is maximally lengthened by dividing of suspensory ligaments, dorsally.

**Figure 4:**
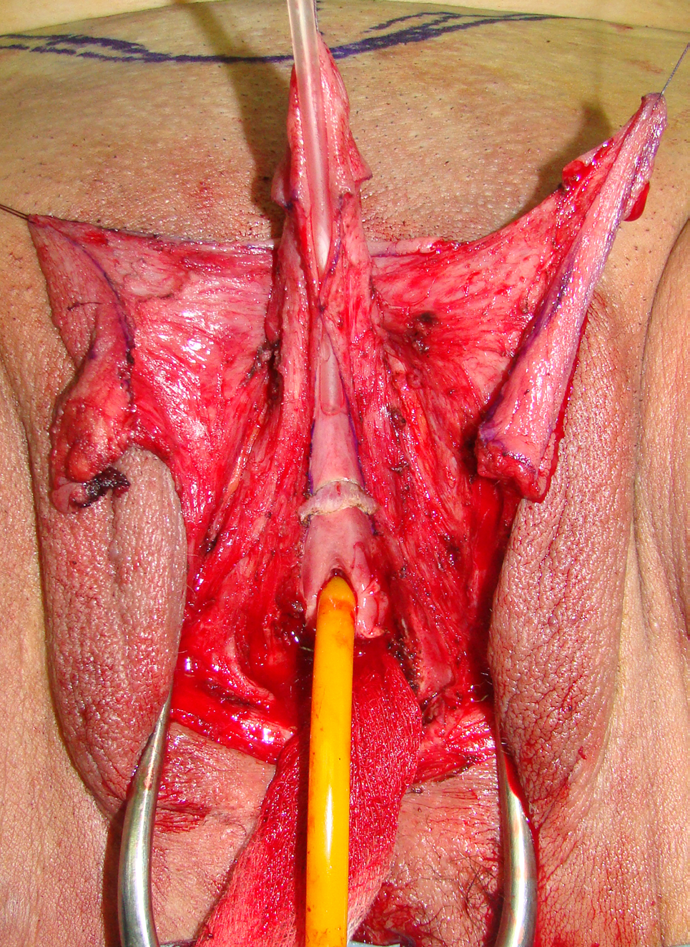
Additional lengthening is achieved by dividing of short urethral plate, ventrally.

**Figure 5:**
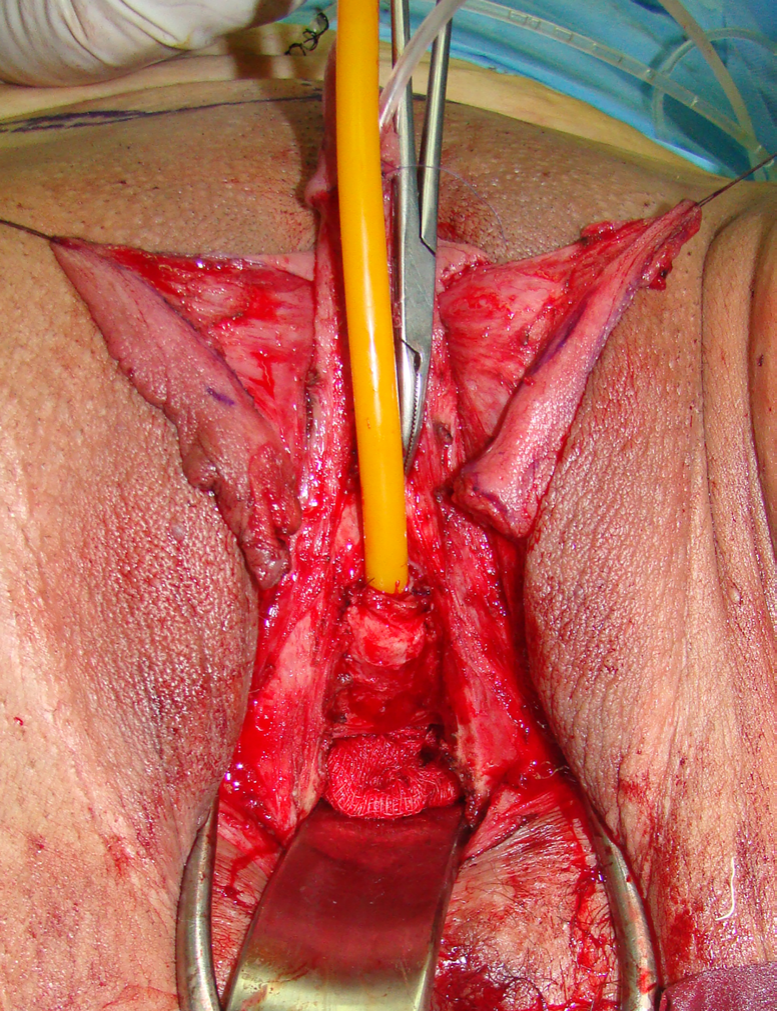
Bulbar part of the neourethra is created and covered with bulbar muscles.

**Figure 6:**
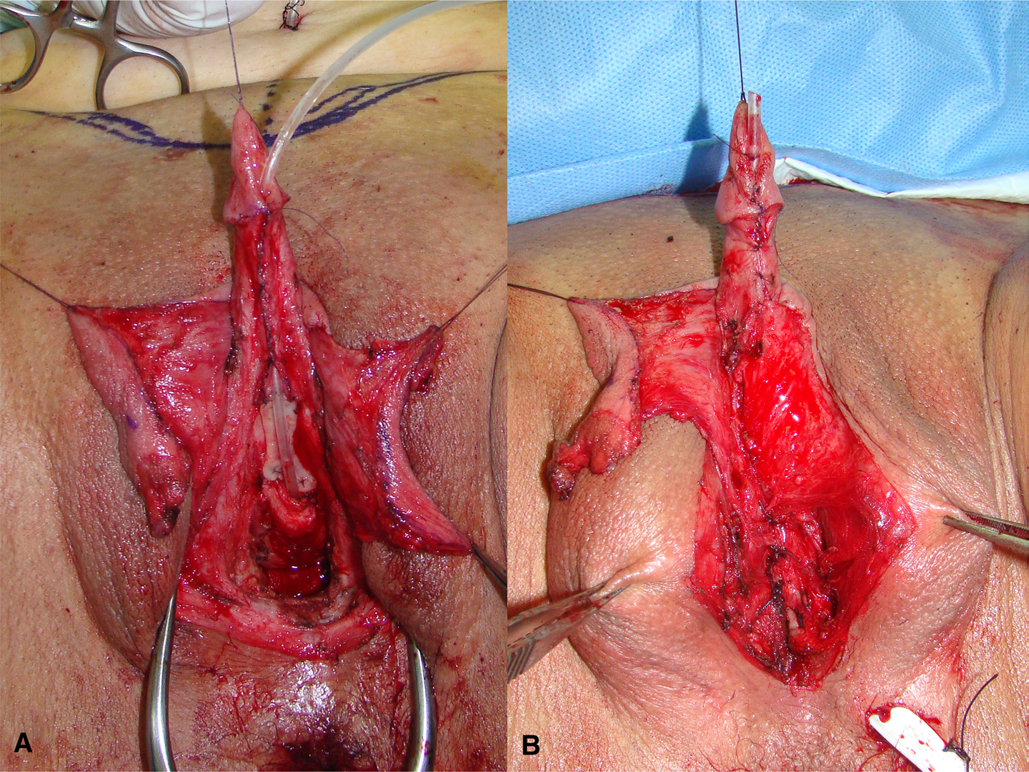
Labia skin graft is fixed in the gap between divided urethral plate **(A)**; left labial skin flap is used for urethral tubularization, while flap pedicle is used for covering of the suture lines; distal urethra is created by simple urethral plate tubularization and glans closure **(B)**.

**Figure 7:**
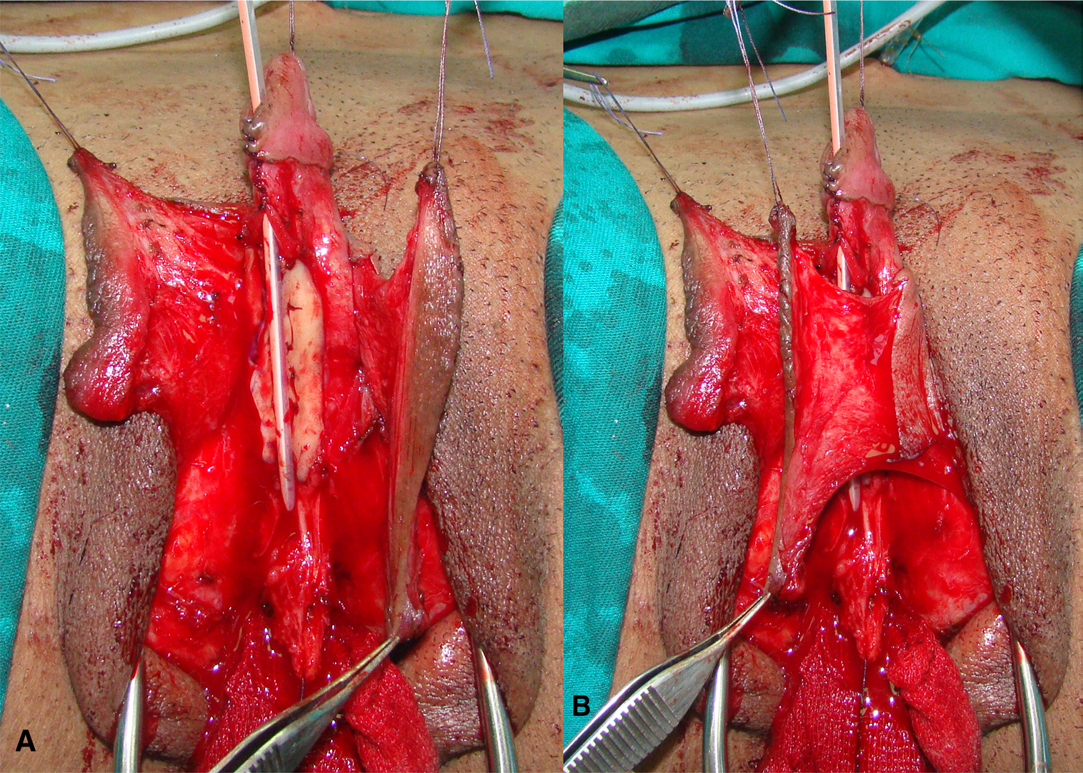
Buccal mucosa graft is used for urethral substitute **(A)**; left labial flap with abundant vascular pedicle is used as an anterior part of the neourethra **(B)**.

**Figure 8:**
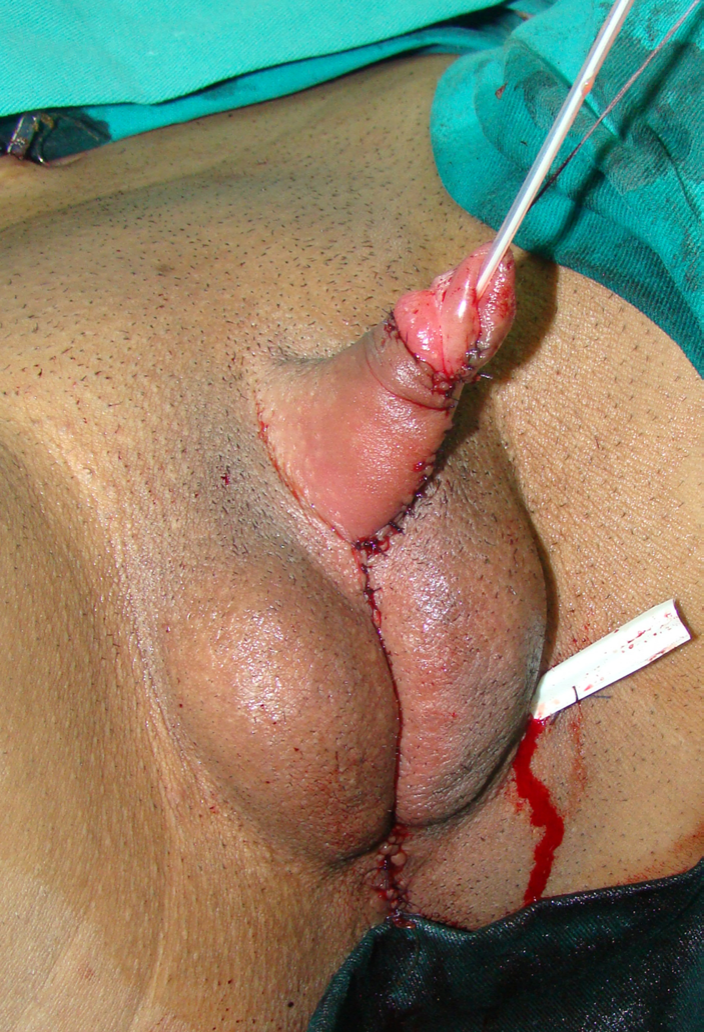
Appearance at the end of surgery. Good size of the neophallus is achieved. Scrotums are created from labia majora and testicular prostheses are inserted.

**Figure 9:**
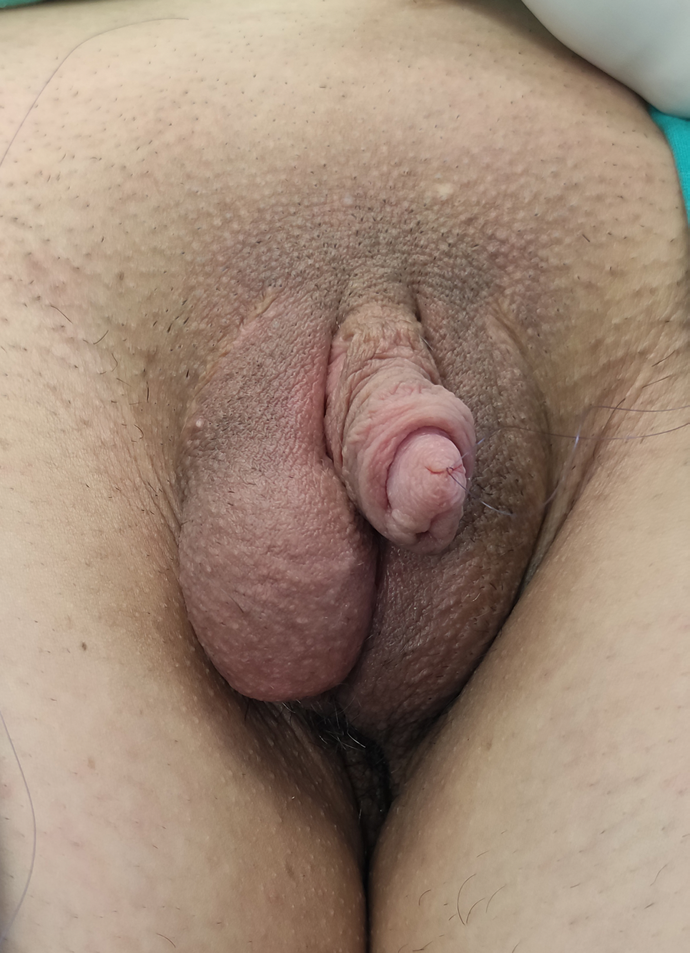
Two years later, good penile length and aestetical outcome are achieved.

The patient is placed in lithotomy position. Elasticated thigh-height stockings and low-molecular weight heparin are used to minimize the risk of deep vein thrombosis. Antibiotic prophylaxis (vancomycin) is administered after anesthesia is introduced. Complete vaginal mucosa is removed by colpocleisis, except one small portion close to the native urethral meatus, which is used later for bulbar urethroplasty. Vaginal vault is completely closed with circular reabsorbable sutures, and male-like perineum is created. A stay suture is placed through the clitoral glans and clitoral degloving is performed by circular incision between the inner and outer layer of clitoral prepuce downwards to the urethral plate, and continued with partial or complete dissection of the suspensory ligaments to lengthen and straighten the clitoris. Additional straightening and lengthening are obtained by urethral plate dissection and division to correct ventral chordee (**Figures 2**–**4**). Careful dissection is needed to prevent the injury of spongy tissue. The most difficult part of the surgery is the urethroplasty which starts with the reconstruction of the bulbar urethra by joining well-vacularized vaginal flap with the proximal part of the urethral plate (**Figure 5**). Additional reconstruction is performed by combining different available flaps and grafts. In cases with a well-developed and long urethral plate, urethral reconstruction can be performed with a simple tubularization of the wide plate, without affecting neophallic length. If a urethral plate was divided, the remaining defect is covered with buccal mucosa graft or labia minora skin graft. Buccal mucosa graft is harvested from the inner cheek by standard technique, with preserving integrity of the buccinator muscle, branches of facial nerve and external orifice of the parotid gland, as well as avoiding cosmetic problems of scarring. Buccal mucosal graft can be replaced by labia minora skin graft in case of good tissue quality or patients preference. This piece of hairless skin graft can be used as an equivalent of buccal mucosa graft (**Figure 6A**). The graft is fixed and quilted to the cavernosal bodies in the gap created after division of the short urethral plate, starting from the advanced urethral meatus to the tip of the glans, thus completing the dorsal aspect of the neourethra. Graft size depends on the neourethral length. Ventral part of the neourethra is created as an onlay flap from the labia minora or a dorsal clitoral skin flap buttonholed ventrally. The inner surface of one of the labia minora is dissected with the dimensions appropriate to cover the dorsal part of the neourethra (**Figure 6B**). Its dissection starts from the vaginal vestibulum and runs upward to the clitoral glans. The lateral wedge of the flap is defined as the border between the inner and outer labial surface. Finally, the flap is harvested by simple de-epithelialization of the outer labial skin, thus perfect vascularization of the flap can be preserved. Then the pedicle is additionally mobilized and lengthened from the subcutaneous tissue of the appropriate labia majora to enable suturing with the dorsal part of the neourethra without tension. The urethra is calibrated to be at least 16 Fr. A 12-14 Fr silicone stent is placed into the neourethra to moisturize inner surface and maintain the urethral lumen. The glans is opened in the midline by two parallel incisions, and both glans wings are dissected extensively to enable glans approxiamtion without tension after urethral reconstruction. Using this technique the neourethral meatus is placed at the tip of the glans (**Figure 7**). Remaining clitoral and labia minora skin is used to cover the penile shaft. It is mandatory to cover all suture lines in order to prevent postoperative urethral complications. Scrotoplasty is done by joining the two labia majora in the midline and silicone testicular prostheses are implanted through bilateral incisions above the labia majora (**Figure 8**). It is very important to create a well-defined penoscrotal angle and male-looking external genitalia (**Figure 9**). In patients with developed mons pubis, additional monsplasty with the resection of the adipose tissue is performed to secure voiding in standing position and create better cosmesis. At the end of the surgery self-adherent dressing is used on the neophallus for 10 days. Suprapubic urinary catheter is maintained for 3 weeks, the urethral stent is removed 7 days after surgery. Vacuum device is recommended for 6 months postoperatively, starting 4 weeks after surgery to prevent adhesions and retraction or shortening of the neophallus.

All patients had a check-up at 3, 6 and 12 months postoperatively. Postoperatively, questionnaires were sent by e-mail including questions about functioning (voiding while standing, erection quality, sensation, possibility of penetration), cosmesis and patients’ satisfaction (18). Satisfaction rates were measured using a 5-point scale (1: totally dissatisfied, 2: mainly dissatisfied, 3: neither dissatisfied nor satisfied, 4: mainly satisfied, 5: totally satisfied).

## Results

The mean follow-up was 94 months (ranged from 16 to 180 months). Mean length of the neophallus was measured 3-6 months after surgery and ranged from 4.8 cm to 10.2 cm (mean 5.6 cm). Mean operative time of metoidioplasty, without additional procedures, was 170 minutes (ranged from 112 to 217 minutes). Mean hospitalization was 3 days (ranged from 1 to 5 days).

Most common complications were related to urethral lengthening (urethral fistula and stricture), and occured in 86 out of total 813 cases (10,55%). Successful result at the 12-month check up was achieved in 86-90% of cases, depending on the type of urethroplasty (**Table 2**). Optimal urethral diameter was confirmed by urethrocystography and uroflowmetry, with an average flow rate of 21.6 ml/sec (ranged from 16.9 to 27.2 ml/sec).

**Table 2:** Urethral lengthening: outcomes and complications.

Type of urethroplasty (groups)	No of cases	Fistula No (%)	Stricture No (%)	Successful cases (%)
**Group A - Tubularization**	92	8 (8.70)	1 (1.10)	83 (90.20)
**Group B - Onlay flap**	42	6 (14.30)	2 (4.75)	34 (80.95)
**Group C - BMG with clitoral skin**	83	9 (10.85)	2 (2.40)	72 (86.75)
**Group D - BMG with labia minora flap**	537	44 (8.20)	8 (1.50)	485 (90.30)
**Group E - labial skin flap/graft**	59	5 (8.50)	1 (1.70)	53 (89.80)
**Total**	813	72 (8.85)	14 (1.70)	727 (89.45)

Other complications included testicular implant rejection in 17 cases (2%) and testicular displacement in 26 cases (3.20%), which was corrected by new implantation or a repositioning of the implant into a proper scrotal pocket. A perineal cyst and/or perineal discharge due to remnant of vaginal mucosa was observed in 78 cases (9.60%). All complications were sucessfully managed with revision surgery 6-12 months after the primary procedure.

Totally 655 patients (80%) answered the questionnaire. According to self-report analysis, majority were satisfied with the cosmetic appearance of their new genitalia (79% totally satisfied, 20% mainly satisfied and 1% dissatisfied). All patients reported good tactile and erogenous sensation of the neophallus. The length of neophallus was inadequate for full penetration in those who reported sexual intercourse. Nevertheless, erection of the clitoris with its completely preserved sensation was reported by all of them. None of the patients reported problems with arousal, masturbation or orgasm.

Seventy six patients (9.35%) required total phalloplasty at our center after metoidioplasty. Musculocutaneous latissimus dorsi free flap phalloplasty was performed in 62, and abdominal phalloplasty in 14 cases.

## Discussion

Neophalloplasty in transmen is one of the most challenging parts of gender affirmation surgery. Since surgical results depend on many factors, including anatomical characteristics, tissue quality and patients’ preference, there is no single and perfect solution for everyone (20). Detailed preoperative consultation with the surgeon and the mental health specialist is necessary in order to discuss the patient’s expectations, available surgical options and complications, moreover to prevent postoperative disappointment and additional surgeries.

Advantages and disadvantages of metoidioplasty, including each technique, have already been well defined (16). Among the currently used metoidioplasty techniques, complete (Belgrade) metoidioplasty is the only one that would enable complete reconstruction and voiding in standing position in a single stage procedure (21). This series presents one of the largest reported series in metoidioplasty, with long-term follow-up. Outcomes regarding cosmetic as well as functional aspect after metoidioplasty are good and quite similar to previous reports (22). However, this study provides a new insight in metoidioplasty from a high-volume center, based on large number of patients, long term follow-up and new urethroplasty modifications.

Reconstruction (straightening and lengthening) of the clitoris remains one of the crucial steps for successful outcome, and is provided by complete division of the suspensory ligaments and urethral plate. Straightening and lengthening of the clitoris significantly increase the length of the neophallus, but its final size depends on the clitoral size before surgery (23). However, this radical approach is not always necessary because it may not result in real or functional gain in penile length, but leads to unpleasing cosmetic results due to the scrotalization of the neophallus. Good appearance of the external genitalia can be achieved by creation of the penoscrotal angle as in a male by using rotational flaps from the remaining clitoral and labia minora skin. This way, residual skin scars are also prevented. Retraction of the neophallus due to scar formation is prevented by postoperative treatment with vacuum pump. For this reason, it is recommended for 6-12 months postoperatively, as an additional factor of good cosmetic outcome. An average neophallic length of 5.6 cm is not different from previous reports. The same is for hospital stay and operative time (18, 23, 24).

Urethral lengthening is the most challenging part of metoidioplasty, with many modifications reported in literature, to achieve better results. Significant improvements were made with using of dorsal clitoral skin flap, button-holed ventrally (17). After division of short urethral plate for clitoral lengthening, incorporating of buccal mucosa graft (BMG) for reconstruction of dorsal urethra became a gold standard, and covered with clitoral skin or labi aminora flap. Combination of BMG and labia minora flap has been proved as the best possible option so far, with low complication rate (23). Lately we have been using labial skin graft instead of BMG, in selected cases. In this way, we preserve buccal mucosa for urethral reconstruction as a part of potential phalloplasty in the future. This urethroplasty technique has a high rate of success in our series (89.80%), almost the same as the technique with BMG (90.30%). Therefore, it can have an important role in patients who are possible candidates for total phalloplasty in the future. One more important issue is the reconstruction of the bulbar urethra. The urinary stream is strongest at the bulbar segment, which therefore is the site with a high risk of fistula formation. Joining the clitoral bulbs over the lengthened urethra, with additional covering using the remaining surrounding vascularized tissue, is considered key to successful fistula prevention (comparison and lengthening). All patients in this series were able to void in standing position, which is one of the main goals of metoidioplasty. This aim is achieved even in obese patients, since obesity is a limitation factor in accomplishing this goal of metoidioplasty. If the neophallus is not long enough, it will stay buried in excessive fatty tissue.

Out of 813 patients in this group, only 86 (10.5%) had urethroplasty complications. All techniques had a success rate of more then 80%, but combination of buccal mucosa graft and labia minora flap should still be considered as the gold standard and primary option. Other complications were mostly cosmetic, and related to testicular implants and vaginal remnants. They are all successfuly treated with minor revision after 6-12 months. It is also important to point out that majority of patients (99%) reported high level of satisfaction with aesthetic appearance after metoidioplasty, without compromising sensitivity and sexual arousal, but without possibility for penetrative intercourse.

A limitations of the study could include a lack of detailed statistical analysis of the cohort, as well as a lack of similar studies in literature for adequate comparison.

The ideal technique for creating neophallus in transmen is still missing. Metoidioplasty, as a single-stage variant of phalloplasty in transmen, is a viable, safe, cost-effective and time-sparing surgical procedure. Good cosmetic and functional outcomes are achieved, with low rate of complications, short hospital stay and high rate of patients’ satisfaction. In patients who present with the desire for total phalloplasty after metoidioplasty, neophallus can be created using either known technique of phalloplasty.

## Data Availability Statement

The original contributions presented in the study are included in the article/supplementary material. Further inquiries can be directed to the corresponding author.

## Ethics Statement

The studies involving human participants were reviewed and approved by Ethics Committee of Belgrade Center for Urogenital Reconstructive Surgery, Approval number: 2020/11). The patients/participants provided their written informed consent to participate in this study. Written informed consent was obtained from the individual(s) for the publication of any potentially identifiable images or data included in this article.

## Author Contributions

NB and MD contributed to conception and design of the study, and NB wrote the first draft of the manuscript. BS and MB organized the database and wrote sections of the manuscript. AS organized the database and revised the final version. All authors contributed to the article and approved the submitted version.

## Conflict of Interest

The authors declare that the research was conducted in the absence of any commercial or financial relationships that could be construed as a potential conflict of interest.

## Publisher’s Note

All claims expressed in this article are solely those of the authors and do not necessarily represent those of their affiliated organizations, or those of the publisher, the editors and the reviewers. Any product that may be evaluated in this article, or claim that may be made by its manufacturer, is not guaranteed or endorsed by the publisher.
